# The Effects of an Intervention Based on the Flipped Classroom on the Learning of Basic Life Support in Schoolchildren Aged 10–13 Years: A Quasi-Experimental Study

**DOI:** 10.3390/children9091373

**Published:** 2022-09-10

**Authors:** Miguel Cons-Ferreiro, Marcos Mecías-Calvo, Vicente Romo-Pérez, Rubén Navarro-Patón

**Affiliations:** 1 Faculty of Education and Sport Sciences, University of Vigo, Campus a Xunqueira, s/n, 36005 Pontevedra, Spain; 2 Facultad de Formación del Profesorado, Universidade de Santiago de Compostela, 27001 Lugo, Spain

**Keywords:** schoolchildren, cardiopulmonary resuscitation, automated external defibrillator, training method

## Abstract

Most out-of-hospital cardiac arrests are attended first by bystanders who are usually friends and/or relatives of the victim. Therefore, the objective of this research was to analyse the impact of a training process based on the flipped classroom on basic life support skills in primary education students. The sample consisted of 308 children (148 experimental group (EG) and 160 control group (CG)) between 10 and 13 years old (M = 10.68 ± 0.64) from 2 schools in Galicia, Spain. The data reveal that the quality parameters are obtained in the number of total compressions in 2 min (CG = 213 and EG = 217; *p* = 0.024) and in the percentage of correct compressions (CG = 87.23% and EG = 91.6%; *p* = 0.013) except for the mean depth and the percentage of correct compressions, which were not reached in any case. Regarding the application of an effective discharge with the Automated external defibrillator (AED), there were no significant differences in the time used by schoolchildren between both methods (*p* = 0.795), but 97.5% (n = 156) of the CG and 100% (n = 148) of the EG are able to do it in just over 1 min. Based on the results obtained, we can conclude that a training program based on the flipped classroom is as effective and viable as traditional training in psychomotricity on CPR techniques and the application of an effective discharge using an AED.

## 1. Introduction

Out-of-hospital cardiac arrest is one of the main health problems in the developed world, causing between 15 and 20% of deaths per year globally [1].

Bystander basic life support (BLS) can improve the chances of survival in the event of sudden cardiac arrest outside the hospital (Riva et al., 2019). Within these BLS are external chest compressions and the early application of defibrillation that can increase the survival of victims by up to 75% [2].

Despite this, in developed countries, immediate resuscitation and early bystander defibrillation are the weakest links in the chain of survival [3]. Therefore, BLS education in early childhood may be key to increasing the effective number of bystanders providing cardiopulmonary resuscitation (CPR) and early defibrillation [4]. In this sense, the World Health Organization (WHO), in agreement with international organizations such as the American Heart Association [5] and the European Resuscitation Council (ERC) [6], recommend initiatives to increase basic CPR by witnesses, which should be taught to all citizens, including schoolchildren, through the so-called “Helping Hands-Training children is training for life” [7]. On the other hand, a strategy to reach the largest possible number of the school population can be to apply this training in educational centers, since their attendance is mandatory, and, in addition, schoolchildren would not only be potential rescuers but also multipliers of CPR knowledge between family and friends [8,9,10,11,12]. Additionally, because learning is taking place in an instructional setting, it is possible to learn attitudes like assisting others, growing in confidence in the success of CPR, developing empathy, and having an internal drive to aid those in need [13,14,15,16,17].

The literature review indicates that schoolchildren are an appropriate population for learning and performing BLS in mannequin simulations [18,19,20]. In addition, it has been confirmed that they learn and maintain this learning for a longer period of time than adults [21]. Among the most debated aspects in relation to training in CPR and defibrillation is the teaching methodology. What is needed is a method that allows for teaching a large number of people in a short time and prevents them from forgetting what they learned as late as possible [22] as well as requiring little time [23]. Several didactic approaches were developed to overcome these problems [24], although there are currently limited data on the ideal teaching method for BLS. The ERC [25] recommends the use of virtual learning environments for pre-skills e-learning, as part of a blended learning approach, or for autonomous learning options.

Despite all this scientific evidence, none of the methods proposed by ERC [25] is feasible for taking to classrooms during the regular 50-minute classroom session in countries like Spain. For this reason, in this study, the training in CPR and AED were carried out using the flipped classroom. The flipped classroom is a teaching method in which the teacher is freed from the time allocated to the presentation of content in the classroom [26] since these are worked on by the students outside the classroom through short videos (pills) [27]. In this way, more time is available to work on the skills of the students in the classroom, which increases the time of interaction and practice of the acquired skills [28,29] and produces greater learning [30]. The justification for the use of this methodology is based on the fact that many of the educational practices carried out in classrooms today are not appropriate to the technological context in which students are immersed, causing low motivation [31]. The flipped classroom is a pedagogical model developed as a result of the inclusion of information and communication technologies in the field of education where it is intended to give a greater role to students in their own learning processes. Proof of this is that the Ministry of Education of the Xunta de Galicia (Spain) launched the E-DIXGAL Plan in 2018 in which each student in the fifth and sixth years of primary education and the first and second years of compulsory secondary education receives a laptop among other things [32].

For all these reasons, the aim of this research was to verify the results of a teaching-learning process in BLS, with the traditional method versus the inverted class, on the skills and sequence of action both in CPR and in the use of the AED and application of an effective discharge.

## 2. Materials and Methods

### 2.1. Study Design

A quasi-experimental design including post-test measurements and a control group was used to perform this research [33].

### 2.2. Participants

Two educational centers in Galicia (Spain) were invited to participate in the research during the academic year 21–22, after completing an ad hoc questionnaire on the previous training received in BLS for their 10–13-year-old students. This age is considered the target population, as indicated by the positioning of the ERC for the teaching of BLS following the recommendations of the “children save lives” proposal [7]. As inclusion criteria, only students who did not have a physical or mental disability that prevented them from learning and performing the basic CPR maneuvers and use of the AED in 5th and 6th grades of primary education could participate in this research. Students who did not participate in the entire process or were admitted to the school after the start of the study were excluded. In addition to this, the informed consent of their parents or legal guardians was necessary to participate in this study.

### 2.3. Tools and Measurements

For the development of the research, the following tools were used and the variables below were collected:

#### 2.3.1. Ad Hoc Questionnaire

The ad hoc questionnaire used was made up of questions related to: (1) sociodemographic data of the student (i.e., name and surnames, age, and sex); (2) previous training received in SVB (yes-no); (3) sequence of action before a person with possible cardiac arrest (1. safety scene; 2. control of consciousness; 3. control of breathing; 4. call the EMS; 5. start external chest compressions) and (4) the correct order AED operating instructions (1. turn on, 2. apply patches, 3. insert patch connector, 4. follow instructions, 5. rinse). Questions (3) and (4) were scored as correct or incorrect according to the answers and the order given by the schoolchildren.

#### 2.3.2. Anthropometric Data

All participating children were measured wearing comfortable clothes and barefoot: weight, height, and through them the body mass index (BMI) calculated using the following equation: weight (Kg)/(height (m))^2^. For this, a scale and a height rod were used. The room used for this part of the study had a comfortable temperature and allowed for the privacy of the participants. The researchers and a member of the management team of the educational center (one member of each gender) were present in the room. The measurements were performed according to the usual protocols and in a homogeneous way in the two schools.

#### 2.3.3. CPR Data Collection

The CPR mannequin used for execution and data collection was the Laerdal Resusci Anne mannequin with Skill reporter software version 2.4 (Laerdal medical AS, Stavanger, Norway), scheduled for chest compressions only.

*BLS Action Sequence:* The action sequence was assessed using an ad hoc checklist, which included each step of the sequence (scene security, consciousness assessment, breathing assessment, emergency call, placement of the hands and initiation of external cardiac compressions). Observers indicated whether or not the step was performed.

*CPR Quality:* The Laerdal Resusci Anne mannequin was used to measure the quality of compression, configuring the parameters according to the ERC 2020 guidelines: chest compression depth of 50–60 mm; compression rate of 100–120 compressions/minute. A threshold of 70% was used as a quality criterion (Perkins et al., 2015 [34]).

#### 2.3.4. AED Data Collection

The Laerdal AED training, a simulation of the Heartstart FR2 + Phillips Defibrillator, served as the source of instruction for AED use. The following variables were collected to record the use of the AED: 1. effective discharge; 2. execution safety; 3. quality discharge; 4. errors made and 5. time to give an effective discharge. Discharge is considered effective if no errors are made that alter the target of the discharge (i.e., chest patch below midline of heart; rib patch below midline of heart; perform the discharge without placing the patches; perform the discharge without pasting the patches or misplaced patches). Safety in execution was considered if the child was not in contact with the mannequin at the time of discharge. A quality discharge was considered if no errors were made, it was done safely, and the execution order was correct: 1. power on; 2. apply patches; 3. insert the patch connector; 4. follow instructions, and 5. discharge.

### 2.4. Procedures

In order to conduct the study, the management of the two educational centres was contacted and informed of the aim of the investigation. The physical education teachers of the various student groups were then contacted. After that, a letter was sent to the parents and/or legal guardians outlining the goal, purpose, design, and procedure of the study (data collection, analysis methods, and their subsequent use), the declaration of confidentiality, voluntary participation, and the freedom to withdraw the child from the study whenever they decide was sent.

**Figure 1:**
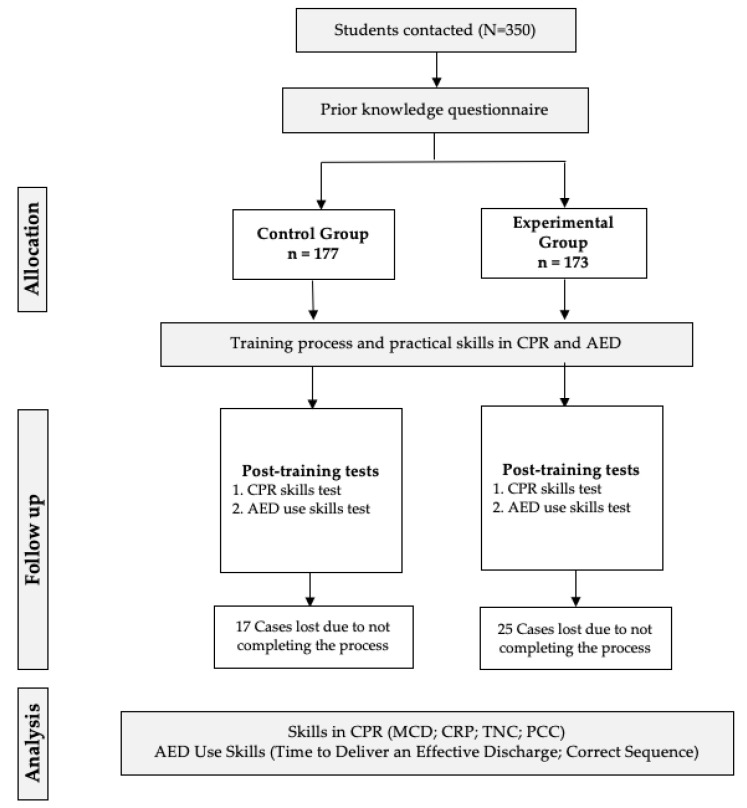
Research flowchart.

Once the signed informed consent of the parents was received, the necessary sociodemographic data (age, sex, height and weight) were recorded. Then, the students were randomized by natural groups (belonging to the same group-classroom and school) to facilitate the training programs development in control group (CG) [traditional training program] and experimental group (EG) [flipped classroom training program]. Before starting the training program, the participating students were given the ad hoc questionnaire to find out their prior knowledge about BLS (Figure 1).

Subsequently, the CG received two 50-min training sessions (100 min in total), one theoretical with instructor-guided training in BLS and AED, during which special emphasis was placed on the importance of performing CPR only hands with uninterrupted compressions and application from the AED; and another practice, with a ratio of an instructor, a CPR mannequin and an AED for every two students. In this, a training was performed assessing the victim including the sequence: safety at the scene, recognition of unconsciousness, opening of the airway and respiratory check, alert to the emergency service, chest compressions, start-up of the AED (CPR only hands for 2 min and the partner applies the AED), repeating this cycle twice per child.

The EG was given the same ad hoc questionnaire as the CG but through the virtual physical education classroom of each school, which they had to complete at home as part of the training method (flipped classroom) so that they had access to 2 short videos: one compression-only external cardiac massage of 3 min and 20 s (https://www.youtube.com/watch?v=ZQdwoRf-TLg (accessed on 10 January 2022).); and another on the use of AED of 3 min 57 s (https://www.youtube.com/watch?v=6W4zbqWWDs20 (accessed on 10 January 2022).), already used in other studies [9,35,36]. The day after this task, the students received the same practical part as the CG under the same conditions (a 50 min session).

Once the training was finished, each student was escorted individually to an isolated room where a simulated scenario was prepared with a Leardal Resusci Anne Q-CPR mannequin programmed in compressions-only mode and an AED. Each participant was asked to act on what they remembered from the training process. When the students started hands-only CPR for 2 min of external cardiac compressions, their parameters began to be recorded. At the end, they were asked to use the AED on the mannequin’s bare chest, recording possible errors in its use and the time it took to deliver a discharge from when they held the AED in their hands until they pressed the discharge button, following the device instructions. Assessments and times were recorded. Two BLS experts evaluated the entire process in real time following the checklists.

The entire process was performed in accordance with the recommendations of the current international cardiopulmonary resuscitation guidelines [37].

### 2.5. Ethics

All research was performed in conformity with the Helsinki Declaration. The University of Santiago de Compostela’s Ethics Committee received the research protocol for approval.

### 2.6. Statistical Analysis

SPSS software (SPSS v.25, IBM Corporation, New York, NY, USA) was used for all statistical analyses. The level of significance was set at *p* < 0.05.

Means and standard deviation were used to express quantitative data’ central tendency, whereas frequencies and percentages were used to express categorical variables. Before the implementation of the training programmes, the differences between the control (CG) and experimental (EG) groups in terms of age, sex, weight, height, and body mass index were assessed using the t test for independent samples.

The chi-square test was used to determine if both groups had similar previous training and action steps for CPR and AED use. Following the application of the training procedure in both groups, a multivariate analysis (MANOVA) was performed for each variable studied in relation to the abilities to perform CPR using only the hands and the time required to apply an effective discharge with the AED. The training programme (conventional vs. flipped classroom) and gender were the two contributing factors (boy vs. girl). The Bonferroni statistic was used to analyse the main effects and the interactions between the variables. Statistical power was expressed using the eta squared statistic (η^2^).

According to the participants’ ages, the average depth of external chest compressions was compared to the anthropometric data using the Pearson correlation analysis. The association was weak, with values between 0.10 and 0.29; moderate, between 0.30 and 0.49; and strong, between 0.50 and 1.00.

The chi-square test was used to assess the differences between groups (CG vs. EG) regarding the sequence of action prior to a person experiencing a potential cardiac arrest and the application of the AED with regard to the effective discharge, safety of the action, or objective quality.

## 3. Results

A total of 308 schoolchildren from 10 to 13 years old participated (M = 10.68; SD = 0.64), with a mean weight of 46.19 Kg (±12.29) and a mean height of 150.63 cm (±12.08). Of these 308 schoolchildren, 69 (44.8%) were girls and 85 (55.2%) boys, 72 (46.8%) in 5th grade and 82 (53.2%) in 6th grade.

The distribution of the participants was 160 in the CG (traditional training) and 148 in the EG (flipped classroom training), respectively.

### 3.1. Baseline Characteristics

Sample baseline characteristics are outlined in Table 1.

The data reflected in Table 1 indicate that the samples of both groups (CG and GE) are similar. The only variable where there are differences is in previous training in BLS, where more than half of the students in the control group claim to have received this training. Even so, if the results of the questions about the action sequence for a possible person in cardiac arrest and the AED action sequence are analysed, it is observed that none of the participants answered correctly. For this reason, it was decided to include all schoolchildren who met the inclusion criteria for this research.

### 3.2. CPR Outcomes

#### 3.2.1. BLS Action Sequence

The results observed in the follow-up of the action sequence before a person with possible cardiac arrest can be seen in Table 2. Scene security (*p* < 0.001, Cramer’s V = 0.273), consciousness assessment (*p* = 0.039; Cramer’s V = 0.117), respiration assessment (*p* = 0.017; Cramer’s V = 0.136), and emergency call (*p* = 0.003; Cramer’s V = 0.168) were the variables between the two groups (CG vs. EG) that showed statistically significant differences. No statistical differences were found in hand placement (*p* = 0.172).

#### 3.2.2. CPR Quality

The mannequin’s parameters can be seen in Table 3.

The findings of the MANOVA show that the training method factor has a significant main effect on the percentage of correct re-expansion, which is higher in the EG but lower than 85% in both groups (F = 3.979, *p* = 0.047; η^2^ = 0.013). There were statistically significant differences in the interaction between gender and training method (F = 4.711, *p* = 0.031; η^2^ = 0.015). Although both boys and girls performed within the recommended parameters (100–120 compressions per minute), statistically significant differences were found in the gender factor regarding the total number of compressions (F = 5.117, *p* = 0.024; η^2^ = 0.017), being the girls of the CG those who performed a greater number of compressions than the boys (*p* = 0.006). Statistically significant differences were also found in the percentage of correct depth (F = 5.732, *p* = 0.017; η^2^ = 0.019), however in neither case was even 4% of the correct percentage reached.

No statistically significant differences between the factors sex, group, or their interaction were found in the remaining factors that were analysed.

### 3.3. AED Outcomes

#### 3.3.1. Results Obtained in DEA Application

A total of 156 participants (97.5%) from the CG and 148 (100%) from the EG (Table 4) simulated an effective discharge (*p* = 0.010), without making any error that prevented it (that is, chest patch below midline of heart; rib patch below midline of heart; perform the discharge without placing the patches; perform the discharge without pasting the patches or misplaced patches). 144 (92.4%) participants from the CG and 148 (100%) participants from the EG were successful in applying a safe discharge (*p* = 0.001) (i.e., without touching the mannequin while the discharge is applied). Finally, of the 304 participants who reached the objective, 148 (92.3%) schoolchildren from the CG and 148 (100%) from the EG also achieved the quality objective, that is, they did not make any mistakes, they did it safely and the order of execution was correct (*p* = 0.001).

#### 3.3.2. Average Times to Apply an Effective Discharge

Table 5 shows the mean times of those schoolchildren who managed to perform an effective discharge (i.e., CG = 156; EG = 148).

The results of the MANOVA performed indicated that there are no statistical differences in the training method factor (*p* = 0.795), or in the gender factor (*p* = 0.086) or in their interaction (*p* = 0.602). Similar results were obtained in the time of application of an effective discharge.

## 4. Discussion

This quasi-experimental study sought to compare the results of two teaching-learning processes in BLS, one through the traditional method (CG) versus the other using the flipped classroom (GE), on the skills and sequence of action, both in the CPR, such as the use of the AED and the delivery of an effective discharge.

There are numerous BLS training methods for schoolchildren, and it has been shown that a 30 min BLS course with mannequins is as effective as classic 4 h courses [38]. Based on the results obtained in this research, the schoolchildren of the GE (flipped classroom method) reach a performance similar to those who received training through a traditional method (CG), both in the sequence of responding to a potential cardiac arrest and subsequent delivery of external chest compressions, such as at the time of delivery of an effective discharge with an AED. It must be taken into account that the traditional learning used with the CG students required several training sessions in which more time was spent than that carried out with the EG, obtaining similar learning results [23]. The learning approach based on flipped learning allowed students to practice or deepen the knowledge and skills learned at home, discussing with the teacher in class and thus have more practical training during class times [24].

Globally analysing the variables studied, in relation to the ability to correctly follow the steps in the event of a possible cardiac arrest, the EG performed better than the CG [39], except in the awareness check (10% correct action). These data corroborate that schoolchildren are a group with a great capacity to learn the steps to follow when faced with a person with possible cardiac arrest [18,19,20]. Although the quality in the execution of the skills is not the most adequate [40,41,42], they could notify the emergency services to request aid.

Regarding the skills to perform basic CPR (Olasveengen et al., 2020 [37]; that is, depth between 5 and 6 cm; frequency of 100 to 120 compressions per minute; complete re-expansion of the chest in each compression performed), the schoolchildren of this study, both of the CG and the EG, does not meet the minimum quality parameters in terms of depth and correct compression percentage.

The percentage of correct depth (number of compressions given with the appropriate depth) only reaches 4% effectiveness in the best of cases, with the objective being 100% [34,37,43]. This could be influenced by the fact that the schoolchildren do not have the age or the anthropometric characteristics necessary to be able to perform a compression at the required depth, as reflected after the results of the correlation applied in this sample between the weight and the average compression depth (Pearson’s coefficient = 0.520; *p* < 0.001), and between the weight and the percentage of correct re-expansion (Pearson’s coefficient = 0.501; *p* < 0.001), indicating that the greater the weight, the greater the average depth [40,41,42]. On the other hand, these results could also have been influenced by the fact that the students did not internalize, during the training, the feedback received from the mannequin, which clearly showed the depth of compression performed by the participants [44]. Despite limitations in percentage of correct depth, it has been shown that students can learn to check scene safety, check for consciousness, assess breathing, call the emergency number, or position their hands correctly during CPR [4], a set of actions that can be very valuable in a real emergency [25].

Just as important as performing external cardiac compression to the correct depth is that the compression is fully released (re-expansion of the chest) so that adequate recoil can occur [37,45]. When comparing the re-expansion of the mannequin’s chest, in both groups (CG vs. EG), in both boys and girls, it is observed that they are close to 100% of the desirable quality recommended by international Resuscitation guidelines [37,45], probably due to the shallow depth of the compressions [46]. Comparing mannequin chest re-expansion by group (CG vs. EG) and gender (boys vs. girls), boys’ outcomes were significantly better (*p* = 0.002) in the EG. All these values regarding the re-expansion of the mannequin’s chest contrast with the previous ones (mean depth and percentage of correct compressions) and seem to indicate that the practical time dedicated to the acquisition of this psychomotricity is adequate [47].

Regarding the mean number of total compressions, they were also similar in the two training groups and in both cases were within the recommendations of the AHA (2020) and the ERC [37]. It can be said that the objective was to achieve 100 external cardiac compressions, and the two groups had a similar maximum average: 106 for the CG and 108 for the EG, which could have influenced the percentage of re-correct expansion [44]. This could be because a more moderate speed allows for better chest recoil than a higher speed, or because the immediate feedback received by QCPR helped students keep average total compressions within the parameters prescribed by the AHA (2020) and the ERC [37].

Regarding the sequence of correct use of the AED, at the beginning of the study none of the participating schoolchildren knew it. This does not mean that students without previous knowledge can perform an effective discharge (this is possible, even if the correct order is not followed) since there are other studies that indicate that a high percentage of children without training could perform an effective discharge [4,46,48]. However, it is important to point out that in this research it was decided not to offer prior knowledge.

Once the training was received, the EG schoolchildren were able to perform an effective quality discharge in 100% of the cases, that is, they did not make any mistakes, they did it safely and in the correct execution order [10]. While in the CG they achieved 92%, even so, these results are higher than those reported in studies with nursing and physiotherapy students [49]; with a degree in Primary Education students [9,50,51,52], with practicing teachers [35,36], with Primary Education students after brief training [46], or with Primary and Secondary Education students without training [48].

Regarding the application of an effective discharge with the AED, we must take into account that, if defibrillation occurs in the first minutes of cardiac arrest, survival rates increase, while for each minute of delay the chances of survival decrease approximately between 10% and 12% [34]. In this sense, we must say that all the schoolchildren who participated in our study and who applied an effective discharge did so in just over 1 min, as in previous research with the same population [46,48]. In this sense, it is worth noting the learning of schoolchildren about the importance of the time factor in the event of cardiac arrest and early defibrillation [48,53], which has led them to complete this procedure promptly.

The limitations found in the development of the study were, firstly, that a quasi-experimental design was used and the group assignments were not completely random for logistical reasons. Second, the results are based on simulation on mannequins, so we do not know how their reactions would be if the schoolchildren had to act in a real situation. Finally, we do not know for how long these knowledge and skills will be maintained since the measurements were made just after the training.

## 5. Conclusions

Our study shows no significant differences between the conventional learning group and the flipped learning group after training. Therefore, we cannot say that flipped learning is superior to conventional learning. However, flipped learning could be comparable with conventional learning.

Flipped learning can be a pedagogical approach that can be used to teach BLS since schoolchildren who received this methodology, to learn CPR and AED skills, have similar results in terms of knowledge and skills.

Among the advantages provided by this methodology are that the time invested in the classroom, for the development of these contents, is less than in traditional teaching, since the lesson directed by the instructor is not carried out with the flipped learning, leaving the practical tasks and the development of skills for the scheduled session [54]. In our study, schoolchildren watch short videos supervised by teachers, studying autonomously. On the other hand, the contents in SVB can be viewed at any time, as many times as they want and at their own pace, to master the content [23].

For all these reasons, a training program based on the flipped classroom is effective, efficient and can be taken to the classroom without modifying the time structure of the teaching sessions (50 min).

## Figures and Tables

**Table 1:** Sample baseline characteristics.

Variable	Control Group (n = 160)	Experimental Group (n = 148)	*p*-Value
Age (years)	10.65 ± 0.71	10.70 ± 0.56	0.474
Gender (male/female)	86/74	84/64	0.596
Weight (Kg)	46.17 ± 13.34	46.22 ± 11.04	0.969
Height (cm)	150.69 ± 14.49	150.56 ± 8.76	0.926
Body mass index (kg·m^−2^)	20.23 ± 4.46	20.28 ± 3.91	0.921
Previous training received (yes/no)	26/134	52/96	<0.001
CPR action sequence (correct/incorrect)	0/308	0/160	1.000
AED action sequence (correct/incorrect)	0/308	0/160	1.000

Note. Data is presented as mean ± standard deviation and frequencies.

**Table 2:** Results for the descriptive analysis of the variables analysed.

		Control Group	Experimental Group
Scene security	No	60 (19.5%)	20 (6.5%)
	Yes	100 (32.5%)	128 (41.6%)
Consciousness assessment	No	12 (3.9%)	22 (7.1%)
	Yes	148 (48.1%)	126 (40.9%)
Respiration assessment	No	6 (1.9%)	0 (0.0%)
	Yes	154 (50.0%)	148 (48.1%)
Emergency call	No	54 (17.5%)	28 (9.1%)
	Yes	106 (34.4%)	120 (39.0%)
Hand placement	Incorrect	2 (0.6%)	0 (0.0%)
	Correct	158 (51.3%)	148 (48.1%)

**Table 3:** Descriptive data of the variables analysed (mean, standard deviation) according to sex and time after training.

		Control Group		Experimental Group	
Variable		M	SD	M	SD
MCD (mm)	boys	28.37	8.3	27.64	8.06
	girls	29.41	9.34	26.63	7.60
	Total	28.85	8.80	27.20	7.86
CRP (%)	boys	85.51	24.94	94.33	7.31
	girls	89.22	18.93	88.84	17.83
	Total	87.23	22.37	91.96	13.19
TNC (2 min)	boys	203.33	52.66	215.31	39.03
	girls	225.27	63.30	219.34	41.11
	Total	213.48	58.58	217.05	39.86
PCC (%)	boys	2.88	7.19	1.88	8.63
	girls	4.43	12.25	0.75	2.39
	Total	3.60	9.86	1.39	6.79

Note. M: Mean; SD: Standard deviation; MCD: mean compression depth; CRP: correct re-expansion percentage; TNC: total number of compressions; PCC: percentage of correct compressions.

**Table 4:** Results for the descriptive analysis of the variables analysed on AED.

		Control Group	Experimental Group
Objective exceeded	No	4 (2.5%)	0 (0.0%)
	Yes	156 (97.5%)	148 (100%)
Security	No	12 (7.7%)	0 (0.00%)
	Yes	144 (92.3%)	148 (100%)
Quality objective	No	12 (7.7%)	0 (0.00%)
	Yes	144 (92.3%)	148 (100%)

**Table 5:** Descriptive statistics of the time variable according to sex and total number of schoolchildren.

Variable		Boys		Girls		Total	
	Group	Mean	SD	Mean	SD	Mean	SD
AED application time (S)	Control	64.95	14.02	63.75	10.39	64.40	12.45
	Experimental	65.61	10.41	62.43	7.68	64.24	9.43

Note. S: Seconds; SD: Standard deviation.

## Data Availability

The data presented in this study are not available in accordance with Regulation (EU) of the European Parliament and of the Council 2016/679 of 27 April 2016 regarding the protection of natural persons with regard to the processing of personal data and the free circulation of these data (RGPD).
